# Knowledge of facts mediate “continuous improvement” in elite sport: a comment on Stanley and Krakauer (2013)

**DOI:** 10.3389/fnhum.2014.00142

**Published:** 2014-03-14

**Authors:** John Toner

**Affiliations:** Department of School of Sport, Health and Exercise Sciences, University of HullHull, UK

**Keywords:** skill, knowledge, expertise, embodiment, neuroscience, conscious processing

Traditional theories of motor skill learning (e.g., Fitts and Posner, 1967) and many contemporary perspectives in sport psychology (see Masters and Maxwell, 2008) and cognitive neuroscience (see Yarrow et al., 2010) argue that skilled action is guided by procedural or implicit knowledge. Researchers adopting this perspective believe that skilled performance proceeds rapidly, efficiently and without the need for conscious monitoring, or a reliance on propositional knowledge (what some might term declarative or explicit knowledge) to guide an activity. However, in a recent paper published in this journal, Stanley and Krakauer (2013) present a compelling argument which offers a stern challenge to the latter of these assumptions. Drawing on evidence from famous cases in neuroscience (e.g., the case of HM) and other research on the nature of skill, Stanley and Krakauer (2013) suggest that becoming proficient at any motor task, and maintaining and improving one's skill in that activity, is heavily influenced by the use of what they refer to as “knowledge of facts” (i.e., propositional knowledge). The aim of this brief commentary is to build on Stanley and Krakauer's work by pointing to empirical evidence (e.g., Collins et al., 1999), recent theory (e.g., Shusterman, 2009) and phenomenological descriptions (e.g., Cotterill et al., 2010) which suggest that “continuous improvement” at the elite level of sport is mediated by the “ongoing accrual and improving application of knowledge of facts about an activity” (Stanley and Krakauer, 2013, p. 2). More specifically, the current paper presents evidence which demonstrates how performers use knowledge of facts in two distinct sporting situations: (1) in the training context when the performer is seeking to improve “attenuated” movement patterns and (2) during the planning and strategizing that occurs in pre-performance routines during on-line competitive performance.

*First*, let us consider how elite athletes might use knowledge of facts to alter and improve movements in the practice context. According to Stanley and Krakauer (2013) the “use of knowledge to select actions continues in the skilled state because there is always the possibility to perform new actions based on further knowledge and then develop acuity at these actions” (p. 7). This appears to be precisely what happens in sport where elite level performers are driven by a desire to learn “new and better techniques” (Breivik, 2007, p. 127). For example, Padraig Harrington, the three-time major Golf champion, explained that he consciously sought to improve his technique throughout his entire career because “what I do every day is keep trying to evolve” (Eden, 2013). Indeed, there is a growing body of empirical evidence to suggest that conscious and deliberate attempts to refine and improve one's movement proficiency are a ubiquitous feature of elite performer's training regimes (see Collins et al., 1999; Ravn and Christensen, in press). Skill refinement might require the performer to draw on knowledge of facts on two occasions. Initially, performers may use “somesthetic awareness” (i.e., “a proprioceptive feel of what they are doing”; see Shusterman, 2009, p. 138) to identify problematic movements during competition. Here, the performer will draw on factual knowledge (which is established with the help of a coach in the practice context) about optimal movement positions and compare it to the kinesthetic feedback they receive from their current movement pattern in the performance context. Next, a coach may use “contrast” drills (see Collins et al., 1999) during training to increase the athlete's conscious awareness and understanding (thereby building on their propositional knowledge) of the correct (desired) vs. incorrect (current) movement positioning.

*Second*, skilled athletes may draw on propositional knowledge when strategizing during the pre-performance routine in closed-skilled sports (e.g., golf). To illustrate, in a naturalistic investigation of the attentional foci adopted by elite golfers during competition, Bernier et al. (2011) found that participants used the pre-shot routine to calculate how certain environmental factors (e.g., the strength of the wind) would influence the shape and trajectory of their tee-shot. Following this, elite golfers may rehearse their swing to establish a “proprioceptive feel” for accurate execution of the stroke (see Nicholls and Polman, 2008). In another study, Cotterill et al. (2010) discovered that elite golfers used the pre-performance routine to identify the risks associated with a particular choice of stroke. Elite golfers appear to bring previous experiences (e.g., those accrued during a practice round) to bear on current problems encountered in the competitive context. Together, these phenomenological descriptions suggest that elite athletes' can retrieve detailed knowledge structures from long-term memory and then re-instate them in working memory to mediate planning and problem-solving during competition. Here performers seem to employ the correct “average action” which, according to Stanley and Krakauer (2013), is “selected from a large potential repertoire… based on ever-accumulating knowledge of the task” (p. 9).

The research literature on the cognitive psychology and neuroscience of skill has tended to present a dichotomized view of the role knowledge plays in guiding skilled performance. According to this body of work, skilled performers engage in intuitive or absorbed coping (see Dreyfus and Dreyfus, 1980) which is predicted to facilitate movement and performance proficiency, or they use declarative knowledge to monitor or control movement which is predicted to have deleterious consequences (see Masters and Maxwell, 2008). Such perspectives appear to represent an unfortunate oversimplification. Indeed, a closer inspection of relevant studies suggests that elite performers are actually quite adept at switching between top-down cognitive processing (i.e., drawing on explicit rules) and bottom-up, embodied feeling and action (i.e., relying on spontaneity) and *vice versa*. This may happen, for example, when they relinquish spontaneity and use knowledge of facts to alter “attenuated” movement patterns in the practice context or when they are strategizing during competitive performance. Although Stanley and Krakauer (2013) use an example of how skilled performers demonstrate *knowledge of what to do to initiate an action*, the current paper provides evidence of how skill may be continually improved by *proprioceptive knowledge of how to change/alter an action* and how performance proficiency may be maintained by propositional *knowledge of how best to plan an action* during competition.
